# Genomic evolution and re-emergence of a multidrug-resistant *Clostridioides difficile* RT027 clone with reduced vancomycin susceptibility driving a prolonged hospital outbreak

**DOI:** 10.1080/22221751.2026.2640707

**Published:** 2026-03-03

**Authors:** Joana Isidro, Filipa Dionísio, Frederico Alves, Soraia Almeida, Cláudia Santos, Manuel Mota Santos, Ernestina Reis, Júlio R Oliveira, João Paulo Gomes, Mónica Oleastro

**Affiliations:** a Genomics and Bioinformatics Unit, Department of Infectious Diseases, National Institute of Health Doutor Ricardo Jorge (INSA), Lisboa, Portugal; b National Reference Laboratory of Gastrointestinal Infections, Department of Infectious Diseases, National Institute of Health Doutor Ricardo Jorge (INSA), Lisboa, Portugal; c Serviço de Prevenção e Controlo de Infeções e Resistências aos Antimicrobianos (SPCIRA), ULS Santo António, Porto, Portugal; d Serviço de Infeciologia, ULS Santo António, Porto, Portugal; e Serviço de Microbiologia, ULS Santo António, Porto, Portugal; f Serviço de Medicina, ULS Santo António, Porto, Portugal; g Animal and Veterinary Research Centre (CECAV), Faculty of Veterinary Medicine, Lusófona University-Lisbon, University Centre, Lisboa, Portugal

**Keywords:** *Clostridioides difficile* hypervirulent RT027 re-emergence, multidrug-resistance, Vancomycin resistance VanR T115A mutation, healthcare-associated outbreak, genomic surveillance

## Abstract

Following an extended period of declining prevalence of the epidemic *Clostridioides difficile* ribotype 027 (RT027) in Portugal, a genetically distinct, multidrug-resistant (MDR) RT027 strain with reduced susceptibility to vancomycin has emerged, causing a 15-month outbreak. This investigation provides epidemiological and genomic evidence for renewed circulation and evolutionary adaptation of this high-risk lineage. A comprehensive outbreak investigation was conducted in a tertiary-care hospital in northern Portugal between 2023 and 2025. Epidemiological and clinical data, antimicrobial exposures, and infection-control measures were analysed. Whole-genome sequencing (WGS) was performed to characterize the outbreak clone. Sixty-six RT027 *C. difficile* infection (CDI) cases were confirmed, with incidence peaking at 5.46 cases per 10,000 patient-bed days in April 2024. WGS revealed an unusual accumulation of AMR determinants conferring a broad MDR phenotype. Notably, all isolates harboured the VanR T115A substitution, a rare mutation previously linked to reduced vancomycin susceptibility, raising concerns regarding evolving antimicrobial tolerance within RT027. The close relatedness to 2016–2018 USA isolates suggests a recent emergence of this clone. Transmission was facilitated by structural constraints, limited isolation capacity and shared sanitary facilities. This prolonged outbreak documents the re-emergence and genomic evolution of a hypervirulent RT027 lineage, characterized by a concerning expansion of antimicrobial resistance and decreased vancomycin susceptibility and a high recurrence rate (25%). These findings highlight the ongoing adaptive potential of *C. difficile* under antimicrobial pressure and underscore the need for strengthened surveillance, genomic monitoring, and infection-prevention strategies to mitigate re-establishment of epidemic RT027 strains in Europe and beyond.

## Introduction

*Clostridioides difficile* is the leading cause of healthcare-associated diarrhoea in Western countries, with the severity of clinical manifestations depending on host characteristics and the infecting strain’s genome makeup [1]. Despite the implementation of stricter infection-control and antimicrobial stewardship practices, CDI remains a significant burden for European healthcare systems, as well as a considerable driver of morbidity and mortality worldwide [2].

The highly resistant nature of *C. difficile* spores challenges infection-control and enables nosocomial transmission via contaminated surfaces. Direct or indirect patient-to-patient transmission, including through healthcare providers, further promotes dissemination contributing to hospital outbreaks [3].

Healthcare-associated (HA) CDI remains predominant, accounting for 65.4% of all CDI cases reported in 2020 [4]. In the early 2000s, the epidemic PCR ribotype 027 (RT027) emerged as the leading cause of HA-CDI in several countries, being associated with outbreaks with higher severity and mortality rates [5]. Selective pressure caused by the extensive use of fluoroquinolones enabled susceptible *C. difficile* strains to acquire resistance determinants, creating conditions that favoured the emergence and spread of epidemic RT027 lineages [6]. However, strict antimicrobial stewardship programs, especially those aimed at reducing fluoroquinolone use, have effectively limited the spread of resistant RT027 strains [7]. Consequently, RT027-associated infections markedly declined, representing only 1.6% of reported CDI cases in the EU from 2018 to 2020 [4].

Current CDI treatment recommendations include vancomycin and fidaxomicin. Although vancomycin remains effective, recent reports of treatment failures linked to emerging resistance [8] are concerning. While still rare, these cases highlight the risk of widespread dissemination of multidrug-resistant clones and the potential emergence of new epidemics [9,10].

This study reports a prolonged nosocomial CDI outbreak caused by a *C. difficile* RT027 clone with an extended antimicrobial resistance profile and reduced susceptibility to vancomycin, in a Portuguese tertiary-care hospital. The outbreak clone was extensively characterized by phenotypic and genomic (including WGS) approaches. Patient-level data were analysed, including treatment regimens, recurrence rates, and the outbreak control measures implemented.

## Materials and methods

### Healthcare setting

This CDI outbreak occurred in a public tertiary hospital in northern Portugal with 674 beds, corresponding to a catchment population of 695,000 people. Written CDI prevention and treatment guidelines have been in place since 2018 and are reviewed every four years by the Infection-Control Team (ICT). Since January 2024, the hospital has participated in the national CDI surveillance network, reporting data and submitting stool samples to the National Reference Laboratory (NRL) according to the European CDI surveillance protocol [11].

### Outbreak data collection

The outbreak was declared on October 12, 2023, and lasted 15 months, ending on January 31, 2025. CDI diagnoses were confirmed by GeneXpert *C. difficile* PCR assay (Cepheid, USA), which targets toxin B (*tcdB*), binary toxin (*cdtA*, *cdtB*), and the *tcdC* deletion characteristic of RT027. Samples presumptively identified as RT027 (n = 38) were sent to the NRL for PCR ribotyping, toxin profiling, antimicrobial susceptibility testing, and WGS.

An outbreak within a ward was defined as three consecutive CDI cases following the last symptomatic episode or the last laboratory-confirmed in the absence of reliable clinical records. All cases meeting the ECDC case definition for HA-CDI [11] and confirmed as presumptive 027 were included. Recurrence was defined as symptom reappearance ≥14 days after the previous episode or, when clinical records were unreliable, after the date of the laboratory diagnosis.

Demographic, epidemiological, and clinical data were collected by the ICT from the hospital chart system and Hepic®. Case severity was classified following ESCMID guidelines [12], and comorbidities were categorized using the McCabe score [11]. The incidence rate was calculated as presumptive 027 CDI associated cases per 10,000 patient bed days.

### Isolation and characterization of *Clostridioides difficile*

A subset of samples identified as presumptive 027 were sent to the NRL for microbiological study (culture, MALDI-TOF identification and antimicrobial susceptibility testing) and genotyping (PCR ribotyping, toxin genes profile and WGS). The detailed description of the procedures is provided in the supplementary material.

### Whole-genome sequencing and genomic analysis

Isolates were processed for more detailed genetic analysis by WGS. Genomic DNA samples were subjected to Nextera XT (Illumina, San Diego, CA) library preparation according to manufacturer’s instructions and paired-end sequenced (2 × 150 bp) on either a NextSeq 550 or NextSeq 2000 apparatus. Genome *de novo* assembly was performed with the INNUca pipeline v4.2.3. Genome annotation was obtained with Prokka v1.14.6 [13].

To assess the genetic relatedness of outbreak strains, a Single Nucleotide Polymorphism (SNP)-based comparative analysis was performed using Snippy v4.5.1, and a maximum-likelihood phylogeny was generated with MEGA. Antimicrobial resistance genes, virulence factors, and mobile genetic elements (MGE) were identified using ABRicate with the ResFinder, CARD, and VFDB databases, and Clostyper. To contextualize the outbreak within the global *C. difficile* diversity, all publicly available ST1 *cdtA+*/*cdtB* *+* genomes were retrieved, quality-filtered, and analyzed using a SNP-based approach with Snippy and Reportree, followed by visualization and cluster identification in GrapeTree. The detailed description of the genomic analysis is provided in supplementary material.

## Results

### Outbreak data description

**Figure 1.**
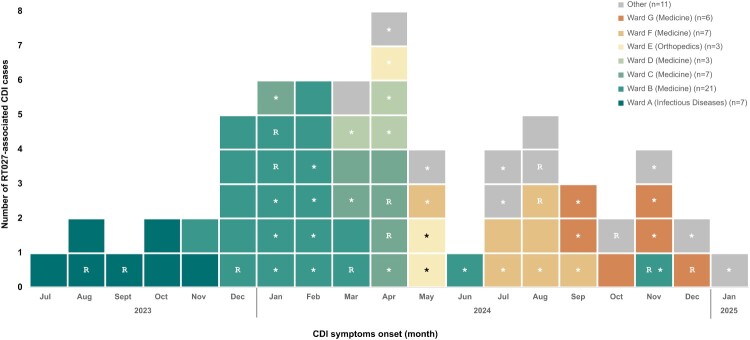
Epidemic curve of RT027 *Clostridioides difficile* infection (CDI) cases, July 2023–January 2025. Distribution of outbreak-associated CDI cases by date of symptom onset. The x-axis shows the time period (months), and the y-axis indicates the number of reported cases. Recurrent cases are marked with “R”, and isolates analysed by WGS are denoted with “*”.

Between October 2023 and January 2025, 64 cases, primarily distributed across seven wards (Table S1), met the outbreak case definition by fulfilling the criteria for HA-CDI cases and were confirmed as presumptive 027. A total of 66 cases were included in this study, comprising 52 first CDI episodes (including two sporadic cases, from July and August 2023) and 14 recurrent cases (one patient had two recurrences) (Figure 1). The average time between the first episode and recurrence was 33 days (ranging from 19 to 60 days). Demographic, epidemiological and relevant clinical data are summarized in Table 1. Notably, all patients (n = 52) had received prior antibiotic therapy within the three months before CDI diagnosis, and 88.5% (n = 46) had taken proton pump inhibitors within the same period*.* Mortality rate was 28.8% (n = 15), with CDI being a contributing cause in six of those cases (11.5%).

**Table 1.** Characteristics of the patients with RT027-associated CDI included in the study.

Patient (n = 52) parameters	n	%
Mean age in years (interquartile range)	77.9 years (72–85.8)	-
Female	31	59.6
Patient origin	**n**	**%**
Home	42	80.8
Nursing Home	7	13.5
Hospital	3	5.8
CDI symptoms prior to hospitalization	11	21.2
Severe CDI	1	1.9
Severe-complicated CDI	4	7.7
Antimicrobial exposure within 3-months before CDI diagnosis	52	100
Proton pump inhibitors intake within 3-months before CDI diagnosis	46	88.5
Comorbidities (McCabe score)	**n**	**%**
Non-fatal	27	51.9
Ultimately fatal	24	46.2
Rapidly fatal	1	1.9

The RT027 CDI incidence rate, considering first episodes and recurrences, rose from zero in January 2023 to 0.7 in July 2023, when the first sporadic case was identified, increasing to 1.34 in October 2023 when the outbreak was declared. In 2024, incidence increased non-linearly, peaking in February at 5.42 and in April at 5.46. The last case occurred in January 2025, and the outbreak was declared over after 56 days without new presumptive 027 CDI cases.

### *Clostridioides difficile* infection treatment and outcomes

The CDI treatment protocol implemented at the hospital follows the updated ESCMID guidelines [12]. However, the heterogeneity of clinical manifestations led to a significant variety in therapeutic options (Table S2). Vancomycin was the most frequently prescribed therapeutic regimen (34/52; 65.4%). In patients treated with vancomycin for 10 or more days, CDI-attributable mortality was 21.4% (6/28), and the first recurrence rate was 39.3% (11/28). Patients experiencing a recurrent infection were treated with either fidaxomicin (76.9%; n = 10) or vancomycin (23.1%; n = 3). During the outbreak period, the overall recurrence rate for RT027 cases was 25% (13/52) and 15.7% (25/159) for non-RT027 cases (Fisher’s exact test, *p* *=* *0.12*).

### Outbreak control measures

Upon detection of a positive CDI result, the ICT notified the responsible clinical team, and reinforced contact precautions, including hand hygiene with soap and water and the use of sporicidal products for cleaning and disinfection. Following the outbreak declaration, periodic reports, audits, and service visits were conducted, with multidisciplinary meetings whenever possible. Formal documentation of recommended measures in patient records was encouraged. Challenges were identified in the selection, donning, and removal of personal protective equipment (PPE), particularly gloves and gowns. Terminal and routine cleaning protocols were reviewed, and the use of sporicidal wipes for high-touch and hard-to-clean surfaces was reinforced. Patients with CDI were placed under contact precautions for *C. difficile* in their respective wards, and non-critical equipment was dedicated to their use. The infection-control measures implemented during the outbreak were maintained beyond the last RT027 case, and this continued vigilance likely contributed to the absence of additional CDI cases.

### Characterization of the outbreak associated clone

All *C. difficile* isolates from outbreak-related samples sent to the NRL (n = 38) were confirmed as RT027. All carried the toxin A (*tcdA^+^*) and B genes (*tcdB^+^*), as well as the binary toxin genes (*cdtA^+^/cdtB^+^*). Regarding antimicrobial resistance, all isolates showed a similar resistant phenotype, showing resistance to clindamycin, erythromycin, gentamicin, moxifloxacin, rifampicin, trimethoprim, linezolid and chloramphenicol, and exhibited an increased MIC to vancomycin (Table 2).

**Table 2.** Antimicrobial susceptibility profile and corresponding resistance determinants of the outbreak *Clostridioides difficile* strain.

Antibiotic	Phenotype (S/R)	MIC (mg/L)	AMR genetic determinants
Clindamycin	R	>256	*ermB*
Erythromycin	R	>256	*ermB*
Gentamicin	R	96–>256	unknown
Imipenem	S	4.0	N.A.
Metronidazole	S	1.5	N.A.
Moxifloxacin	R	>32	GyrA T82I
Rifampicin	R	>32	RpoB R505K
Tetracycline	S	0.094	N.A.
Trimethoprim	R	>32	*dfrF*
Linezolid	R	6	*cfr(B)*
Chloramphenicol	R	32–48	*cfr(B)*
Vancomycin	S*	2.0	VanR T115A

MIC – Minimum Inhibitory Concentrations.

S – Susceptible.

S* Reduced susceptibility (S ≤ 2,0; R > 2,0).

R – Resistant.

N.A – Not applicable.

Note: Metronidazole MICs were determined for surveillance purposes, but although the isolates were susceptible *in vitro*, this antibiotic was not used as single-drug therapy, as current guidelines favour vancomycin or fidaxomicin due to higher efficacy and lower recurrence. Fidaxomicin MICs were not determined, as clinically relevant resistance in *C. difficile* is extremely rare and routine testing is not recommended by current treatment guidelines.

### WGS data analysis

**Figure 2.**
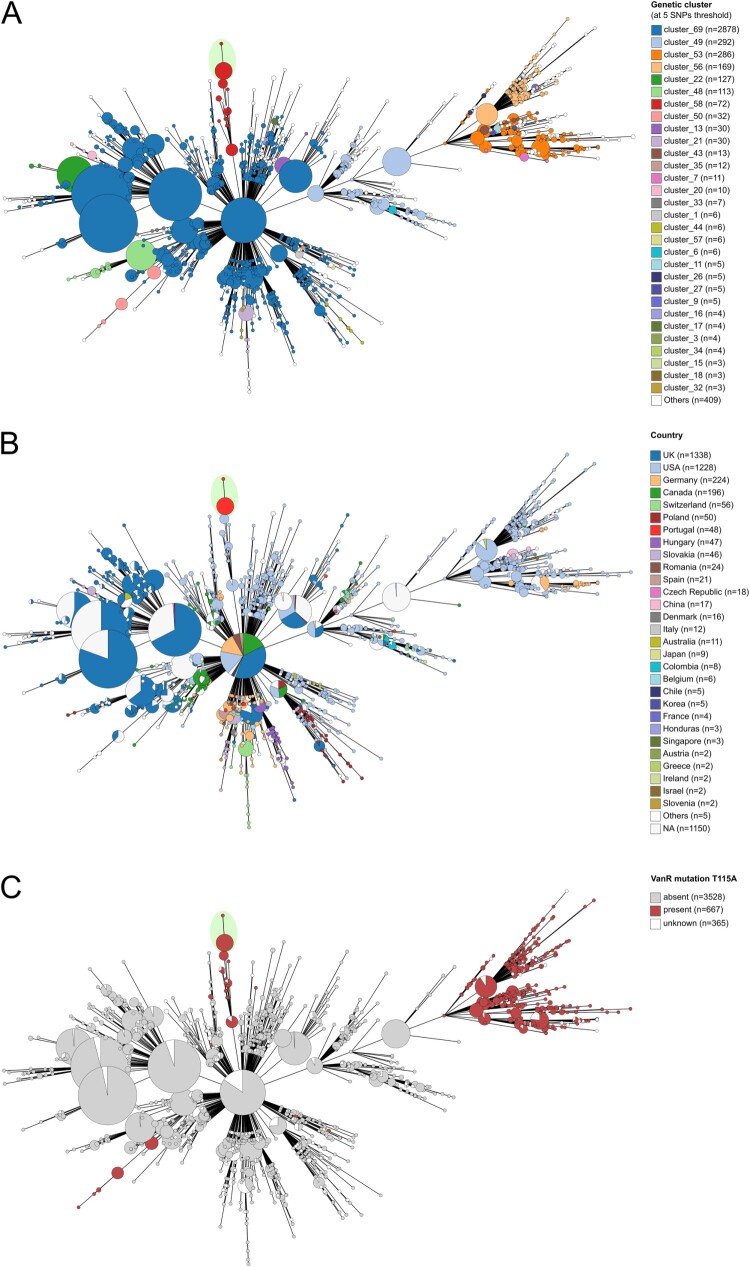
Minimum spanning tree (MST) of a global dataset of RT027 genomes. The MST was generated with Reportree based on 8065 core variant positions extracted from a multiple genome alignment of 4560 sequences with 4191339 bp generated with Snippy-core after mapping against the *C. difficile* strain R20291 reference genome (see methods for details). The MST was visualized in Grapetree with branches collapsed at 1 SNP threshold. MST nodes are coloured according to A) the genetic clusters identified by Reportree at a threshold of 5 SNPs, showing the Portuguese outbreak-associated sequences (highlighted by a green ellipse in both panels) clustering with USA sequences (cluster_58), to B) the country where the isolates were collected, and C) the presence of the VanR mutation T115A.

**Figure 3.**
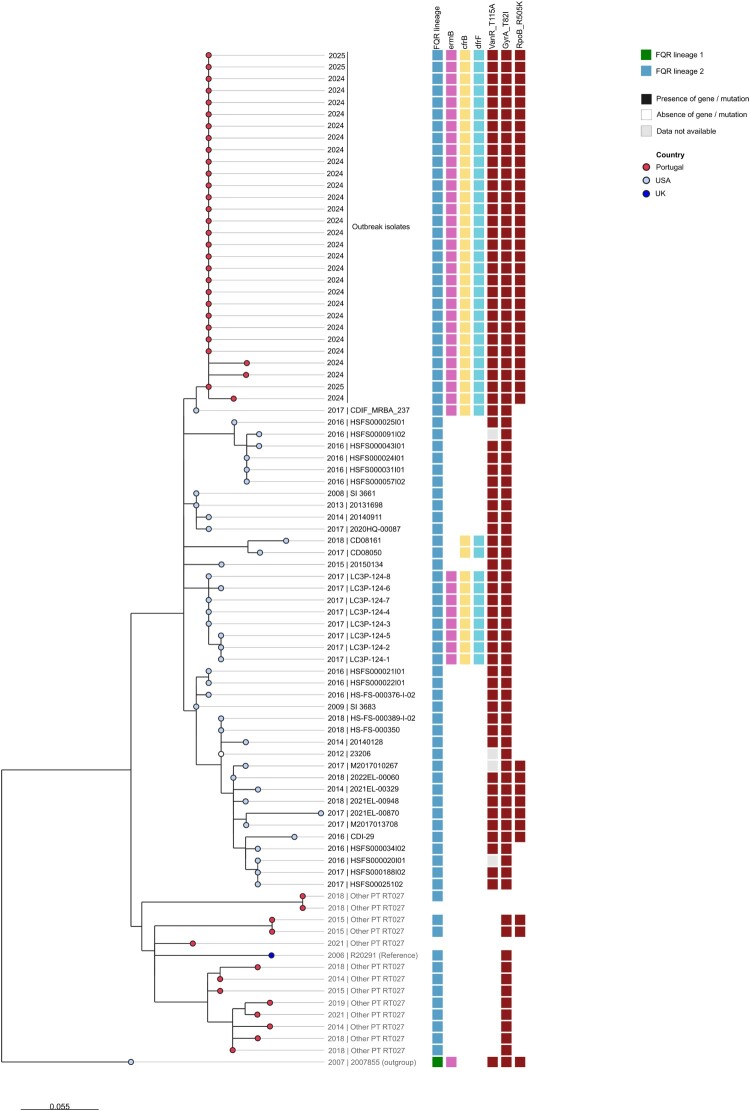
Zoom-in phylogenetic analysis of the genetic cluster with the genomes most closely related to the outbreak clone in the global dataset analysis. Maximum-likelihood tree (with 100 bootstraps) of 30 outbreak-associated genomes and 42 genomes closely related to the outbreak clone (genetic cluster at 5 SNPs threshold on the global dataset analysis). For phylogenetic context, 13 other Portuguese RT027 genomes (not related to the outbreak), the reference genome R20291, and a genome from lineage FQ-R1 (strain 2007855, acc. no. GCF_000210455.1) (outgroup) were also included in the analysis. The phylogeny was based on 162 core variant positions, extracted from the full alignment returned by Snippy variant caller after mapping of sequence data against the reference genome R20291 (FN545816.1). The tree leaf nodes are coloured according to the country of origin and the labels indicate the year of collection and strain ID. Coloured metadata blocks indicate the fluoroquinolones resistance (FQR) lineage and the presence/absence of determinants of resistance in each genome. Of note, three of the 33 outbreak-related genomes were not included in this analysis due to the exclusion criteria of the upstream global clustering analysis (see details in methods).

A subset of 33 isolates was selected for WGS, encompassing all affected wards and spanning the entire sampling period from January 2024 to January 2025. *In silico* MLST confirmed all as ST1, carrying *tcdA/tcdB* and *cdtA/cdtB*. SNP analysis showed high clonality, with most genomes being identical (mean distance of 1 SNP) (Figure 2A). Resistance genes detected in all isolates included *ermB* (MLSb-resistance), *cfrB* (resistance to antibiotics targeting the 23S rRNA), *dfrF* (trimethoprim resistance), *cdeA* (a multidrug transporter), and *blaCDD-2* (beta-lactam resistance). Key mutations found were *gyrA* 245C > T (GyrA T82I; fluoroquinolone (FQ) resistance), *rpoB* 1514G > A (RpoB R505 K; rifampicin resistance), and *vanR* 343A > G (VanR T115A; reduced vancomycin susceptibility) (Table 2; Figure 3) [10].

### Mobile genetic elements

Since resistance genes in *C. difficile* are often MGE-associated, genomes were screened for transposons and plasmids. On all isolates, the *ermB* gene was located on a ∼20 kb Tn6189-like element (97.9% identity, 93% coverage; MK895712.1) inserted upstream of the locus *CDR20291_1081* (Figure S1). The *dfrF* gene occurred within a 6.5 kb integrase–excisionase region inserted between *CDR20291_0628* and *CDR20291_0629* (Figure S2). BLASTn showed this element in other *C. difficile* strains (e.g. CP126076.1 and CP149711.), although in a different chromosomal location, and also in other bacterial species, such as in an *E. faecium* plasmid (AP026581.1), attesting to its mobility. The *cfrB* gene was embedded in a ∼10.5 kb MGE between *CDR20291_2866* – *CDR20291_2867*. This MGE is distinct from the known *cfrB*-containing Tn6128 transposon and likely derived from an *E. faecium* transposon (Figure S3). Additionally, the 58 kb transposon Tn6110 (BK008009), carrying a *rlmN*-type 23S rRNA methyltransferase associated with macrolide resistance in *C. difficile* [14], and plasmid pCD-ECE3 (2.8 kb; LR594543.1) were present in all isolates.

### Context of the outbreak clone in the global genomic diversity

The outbreak-associated *C. difficile* genomes were integrated into the global genetic diversity of RT027, using a dataset of 4527 publicly available RT027 genomes (Table S3) and 13 additional outbreak-unrelated RT027 genomes from Portugal. The outbreak-associated isolates belong to the more globally spread FQ-R2 lineage, and are closely related to a group of USA strains (Figure 2B and Figure 3). Regarding the distribution of the determinants *ermB*, *cfrB* and *dfrF*, the outbreak sequences, together with the above-mentioned USA genomes and two unrelated sequences, were the only ones found to harbour the three genes simultaneously, highlighting the unique profile of this clone. Regarding the VanR mutation T115A, we observed that it is highly prevalent among FQ-R1 isolates, potentially representing an ancestral SNP in this group, but it only appears in a few genetic clusters in the FQ-R2 lineage, including the one containing the outbreak genomes and this same subset of USA sequences (Figures 2C and S4).

To accurately identify the sequences in the global dataset most closely related to the outbreak genomes, genetic clustering data obtained with Reportree at different SNP thresholds were evaluated. At thresholds ranging from 0–4 SNPs, the outbreak sequences formed a distinct group; at the threshold levels of 5–8 SNPs, the outbreak sequences consistently clustered with a subset of 41 USA sequences, most of which collected between 2016 and 2018 (Figure S5 and Figure 2B).

A new phylogenetic analysis was then performed over this subgroup of closely related sequences (Figure 3), 13 other RT027 sequences from Portugal unrelated to the outbreak, the reference genome of strain R20291 and an outgroup genome from FQ-R1 (strain 2007855, acc. no. GCF_000210455.1). The genome of the USA strain CDIF_MRBA_237, is the one most closely related to the outbreak genomes, at a 1 SNP distance from the outbreak root, and harbours a highly similar resistant genotype, only missing the RpoB R505 K mutation. The same was observed for a group of eight strains collected in the USA also in 2017, LC3P-124-1 to LC3P-124-8, which collectively potentially point to the origin of the outbreak lineage.

### Within-outbreak genetic diversity

**Figure 4.**
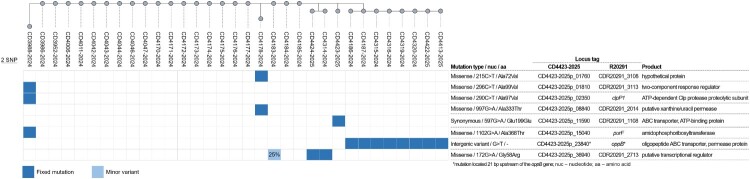
Genetic variability and microevolution of the outbreak clone. Neighbour-joining phylogenetic tree of the 33 outbreak-associated genomes based on 8 core variant positions. To maximize the number of informative genomic sites, a representative genome from the outbreak dataset (CD4423-2025) was used as reference for mapping. The panel below the phylogenetic tree shows the presence/absence of the identified mutations among the outbreak genomes and the table on the right describes the mutations types and effects, locus ID and product annotation. The locus ID are indicated for both isolate CD4423-2025 and for the homologous genes on the reference genome of strain R20291 (acc. no. FN545816.1). The proportion of the population with a given minor variant is shown on the corresponding mutation block (light blue) in the presence/absence panel. Fixed mutation (dark blue) – mutation present at a proportion of ≥90% in the population. Minor variant (light blue) – mutation present at a proportion of ≤50% in the population.

A fine-tuned analysis of the mutations of all the outbreak-associated genomes was then performed using one of the isolates (CD4423-2025) as reference. Twenty of the 33 genomes, from cases occurring in months with the highest incidences, had the same exact profile and hence presented no mutations to the outbreak root (Figure 4). Three genomes presented singleton profiles with 1–3 SNPs, i.e. the mutations were exclusive to each sequence (CD3988-2024, CD4178-2024 and CD4423-2025). Notably, the three SNPs found in the CD3988-2024 isolate, from January 2024, were all non-synonymous and located in distinct genes: *rgaR* (two-component system response regulator), *purF* (amidophosphoribosyltransferase), and *clpP1* (ATP-dependent Clp protease proteolytic subunit) (Figure 4). Two isolates from the same patient, first episode (CD4314-2024) and recurrence (CD4424-2025) shared a missense mutation in a putative transcriptional regulator, previously detected as a 25% minor variant in CD4183-2024, indicating mutation emergence and a likely shared transmission chain. Furthermore, another cluster of eight isolates shared a mutation in an intergenic region, found 21 bp upstream of *oppB* operon.

## Discussion

The emergence of hypervirulent FQ-R *C. difficile* RT027 lineages has reshaped the epidemiology of HA-CDI. Initially identified in North America, where it caused major outbreaks in Canada [15] and the USA [16], RT027 rapidly disseminated to Europe [17], driven by the acquisition of the FQ-R mutation T82I in the *gyrA* gene [18] by two distinct lineages, FQ-R1 and FQ-R2, which arose independently under selective pressure from extensive fluoroquinolone use [19].

These epidemic lineages demonstrated enhanced transmissibility and virulence, leading to widespread outbreaks with increased morbidity and mortality [20]. The implementation of stricter antimicrobial stewardship, particularly restrictions on fluoroquinolones, contributed to marked declines in RT027 prevalence across Europe over the past decade, demonstrating the effectiveness of targeted antibiotic policies in controlling epidemic strains [7,21].

The outbreak described here stands out as a notable deviation from this downward trend. In Portugal, the prevalence of FQ-R RT027 declined from 24.3% in 2014 to below 0.5% by 2016 [22], with no reported cases in 2022–2023 (NRL unpublished data). This 15-month outbreak involved 66 cases and was characterized by severe clinical outcomes, including a CDI-attributable mortality rate of 21.4% and a recurrence rate of 25%, consistent with reports from RT027 outbreaks in other countries [23], and considerably higher than those observed for infections caused by other RTs [24]. This outbreak illustrates the capacity of epidemic clones to re-emerge in healthcare settings, even after prolonged periods of apparent absence, highlighting the ongoing threat posed by hypervirulent *C. difficile* lineages.

Transmission dynamics were influenced by multiple factors. Structural constraints, including limited access to single-room isolation, and challenges in the appropriate use of PPE, likely facilitated patient-to-patient spread. The outbreak persisted despite reinforcement of infection-control measures, emphasizing the necessity of bundled interventions that simultaneously address environmental decontamination, hand hygiene, contact precautions, and targeted staff training. These findings align with previous evidence that integrated, multi-component strategies are generally more effective than single interventions in controlling HA-CDI outbreaks, particularly in high-risk healthcare environments [23,24].

Antimicrobial stewardship is a cornerstone of CDI control [25]. The outbreak clone’s extensive antimicrobial resistance profile underscores the importance of limiting unnecessary antibiotic exposure [24]. While vancomycin therapy was consistent with international guidelines [12], its routine use may have contributed to the prolonged infection course, especially in the context of the VanR T115A mutation identified in all outbreak isolates. Indeed, patients receiving single-drug vancomycin therapy had a 39.3% recurrence rate. Disease persistence despite vancomycin therapy illustrates the clinical challenges posed by emerging MDR clones. Given that hypervirulent, epidemic, healthcare-associated strains such as RT027 are linked to higher recurrence rates than community-associated strains [2], these findings underscore the importance of prioritizing fidaxomicin as first-line monotherapy, particularly for RT027-associated CDI. Hospital-level stewardship interventions, including restriction of broad-spectrum antimicrobials, remain critical, particularly when integrated into bundled strategies alongside infection-control measures.

WGS confirmed clonality of the outbreak strain, with minimal SNP variation consistent with a healthcare-associated outbreak [26]. Interestingly, in March 2025, this clone was isolated from two patients with fulminant HA-CDI in another hospital in northern Portugal (data not shown), with no known links to the original outbreak.

The outbreak clone exhibited an unusual accumulation of antimicrobial resistance determinants (Table 2), including the rare *C. difficile* VanR T115A substitution [10]. Although this mutation has been associated with reduced vancomycin susceptibility, its *in vivo* implications, including potential impacts on treatment failure, remain unclear. In addition, faecal concentrations of vancomycin have been shown to be substantially higher than the MICs measured *in vitro* for *C. difficile* isolates, creating uncertainty regarding the clinical significance of increased MIC values [27]. The clone also carried the uncommon *dfrF* and *cfrB* genes, conferring resistance to trimethoprim, linezolid, and chloramphenicol. These genes are rarely reported in *C. difficile* [28,29] and are typically associated with mobile genetic elements in *Enterococcus faecium*, highlighting the potential for interspecies gene transfer and the genomic plasticity of *C. difficile*. While no single resistance determinant fully explains the observed clinical outcomes, the combination of multiple genomic traits may influence transmissibility, virulence, and treatment response, reflecting a broader trend of multidrug resistance as a characteristic feature of epidemic lineages [6].

Additional genomic observations suggest ongoing adaptive evolution during the outbreak. Notably, several later isolates harboured a mutation upstream of the *opp* operon (Figure 4), whose disruption is known to lead to increased sporulation, affecting persistence and virulence [30]. Although further studies would be needed to assess the effect of these particular mutations, their retention across multiple wards suggests potential selective advantages. None of the identified mutations were associated with known resistance mechanisms. Accordingly, no changes in the resistance phenotype of the clone were observed over time, indicating that the therapeutic modifications applied during the outbreak did not translate into detectable genotypic or phenotypic resistance. Additionally, phylogenomic analysis indicated close relatedness to 2016–2018 USA RT027 isolates, with a ≥ 5 SNPs distance (Figure 2A), which, being consistent with the expected evolution of *C. difficile* over this timespan [31], suggests a recent intercontinental dissemination. Nonetheless, despite sharing a highly similar genotype, the closest USA isolates lack the RpoB R505 K mutation (Figure 3), which might have emerged later as an adaptive response to rifampicin selective pressure. A recent meta- analysis reports that resistance to rifampicin is not randomly distributed among *C. difficile* ribotypes but is significantly associated with certain types, including RT027 (≈47% resistance), indicating that rifamycin class antimicrobial pressure can select for resistant clones in clinical settings [32]. The uncommon occurrence of VanR T115A within the FQ-R2 lineage further indicates independent and sequential adaptation under combined fluoroquinolone and vancomycin pressure, illustrating the dynamic evolutionary potential of epidemic clones (Figures 2C and S4).

Clinical outcomes in this outbreak were influenced by both strain characteristics and institutional factors. High-risk patient populations, including oncology, transplant, and intensive care units, contributed to broad-spectrum antibiotic exposure and increased vulnerability to infection. Structural limitations, including the availability of single rooms and shared sanitation facilities, constrained the effectiveness of infection-control measures. Treatment variability, including inconsistent duration of therapy and frequent antibiotic changes, likely compounded challenges in managing CDI. These findings underscore the need for both optimized antimicrobial therapy and structural support to enhance infection-control and reduce recurrence during outbreak events.

The outbreak also highlights the broader implications of emerging MDR RT027 clones. Beyond immediate hospital-level concerns, the potential for inter-hospital and international dissemination is substantial, given the documented genetic relatedness to isolates from the USA. The combination of hypervirulence, multidrug resistance, and genomic plasticity increases the risk of re-establishment of epidemic lineages even in regions where RT027 prevalence had previously declined. This emphasizes the critical role of continuous genomic surveillance to detect early re-emergence, identify novel resistance determinants, and guide both infection-control and therapeutic strategies.

From a public-health perspective, the findings reinforce several key principles. First, MDR clones can persist and re-emerge despite prior declines, particularly in settings with structural or operational vulnerabilities. Second, genomic characterization provides essential insights into adaptive evolution, resistance acquisition, and potential interspecies gene transfer, informing targeted interventions. Third, integrated infection-control strategies combined with antimicrobial stewardship are necessary to control outbreaks, prevent sustained transmission, and mitigate recurrence. Finally, therapeutic approaches may need to be tailored in the presence of emerging resistance, including consideration of agents such as fidaxomicin to reduce recurrence in high-risk clones.

In conclusion, this prolonged outbreak documents the re-emergence and genomic evolution of a hypervirulent, multidrug-resistant RT027 lineage in Portugal. The rare VanR T115A mutation, extended resistance profile, and adaptive mutations in sporulation and virulence-associated loci underscore the evolutionary potential of epidemic *C. difficile* strains. The outbreak highlights the interplay between pathogen characteristics, antimicrobial pressure, and healthcare environment factors in driving sustained transmission. These findings emphasize the need for ongoing genomic surveillance, optimized antimicrobial stewardship, and robust infection-prevention strategies to prevent the re-establishment of epidemic RT027 clones in Europe and beyond.

## Supplementary Material

Figure S3.png

Figure S2.png

CDI MDR RT027 Outbreak_PT_Supplementary material_revised.docx

Figure_S5.png

Figure_S4_new.png

Figure S1.png
