# Supplementary figures and images for: Genomic evolution and re-emergence of a multidrug-resistant Clostridioides difficile RT027 clone with reduced vancomycin susceptibility driving a prolonged hospital outbreak
Source: Emerg Microbes Infect. 2026 Mar 3;15(1):2640707. doi: 10.1080/22221751.2026.2640707 (PMC12997477; doi:10.1080/22221751.2026.2640707)

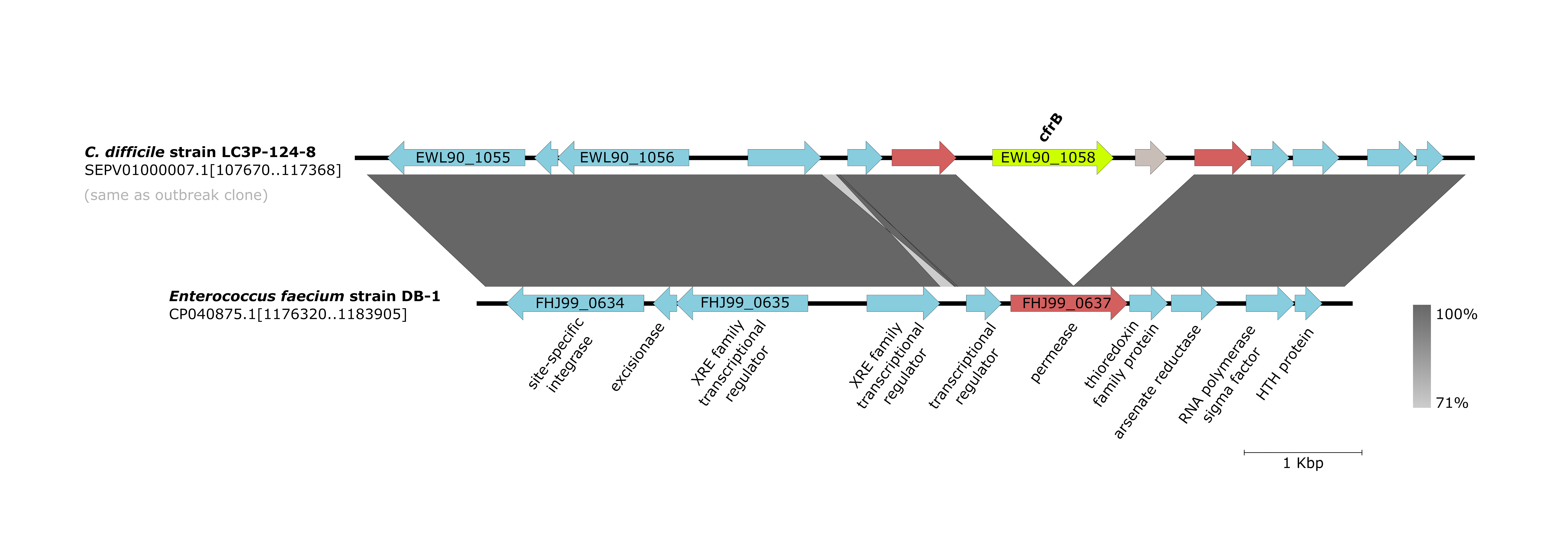

Supplement: Figure S3.png [file TEMI_A_2640707_SM1126.png]

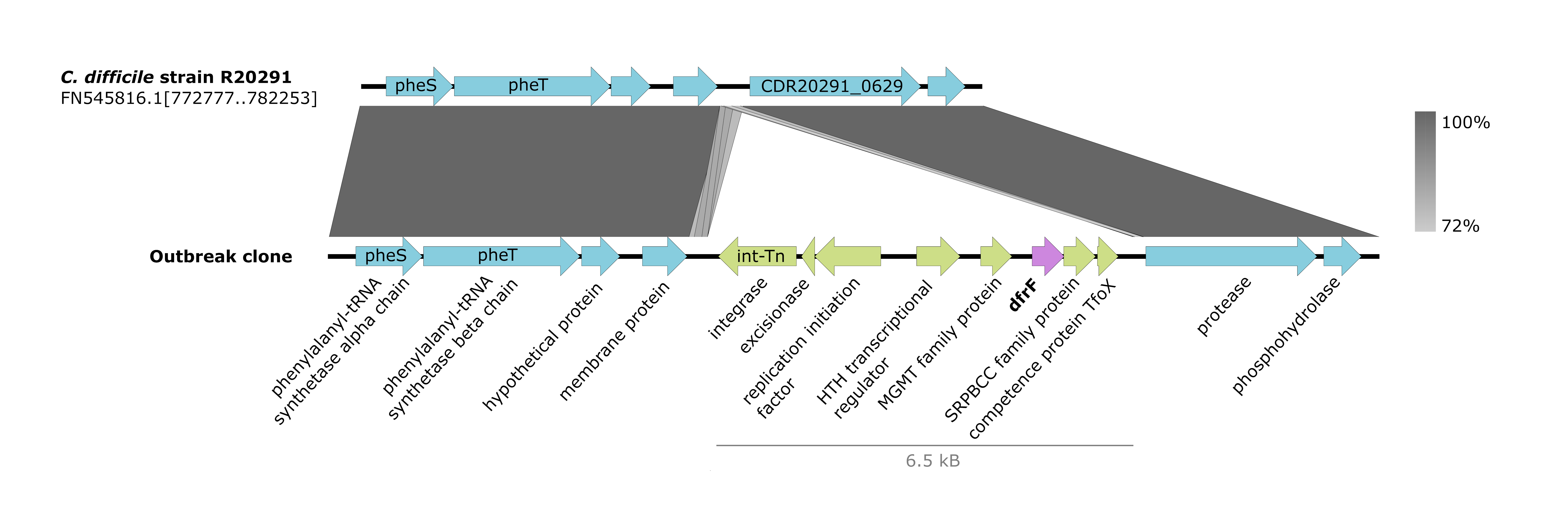

Supplement: Figure S2.png [file TEMI_A_2640707_SM1125.png]

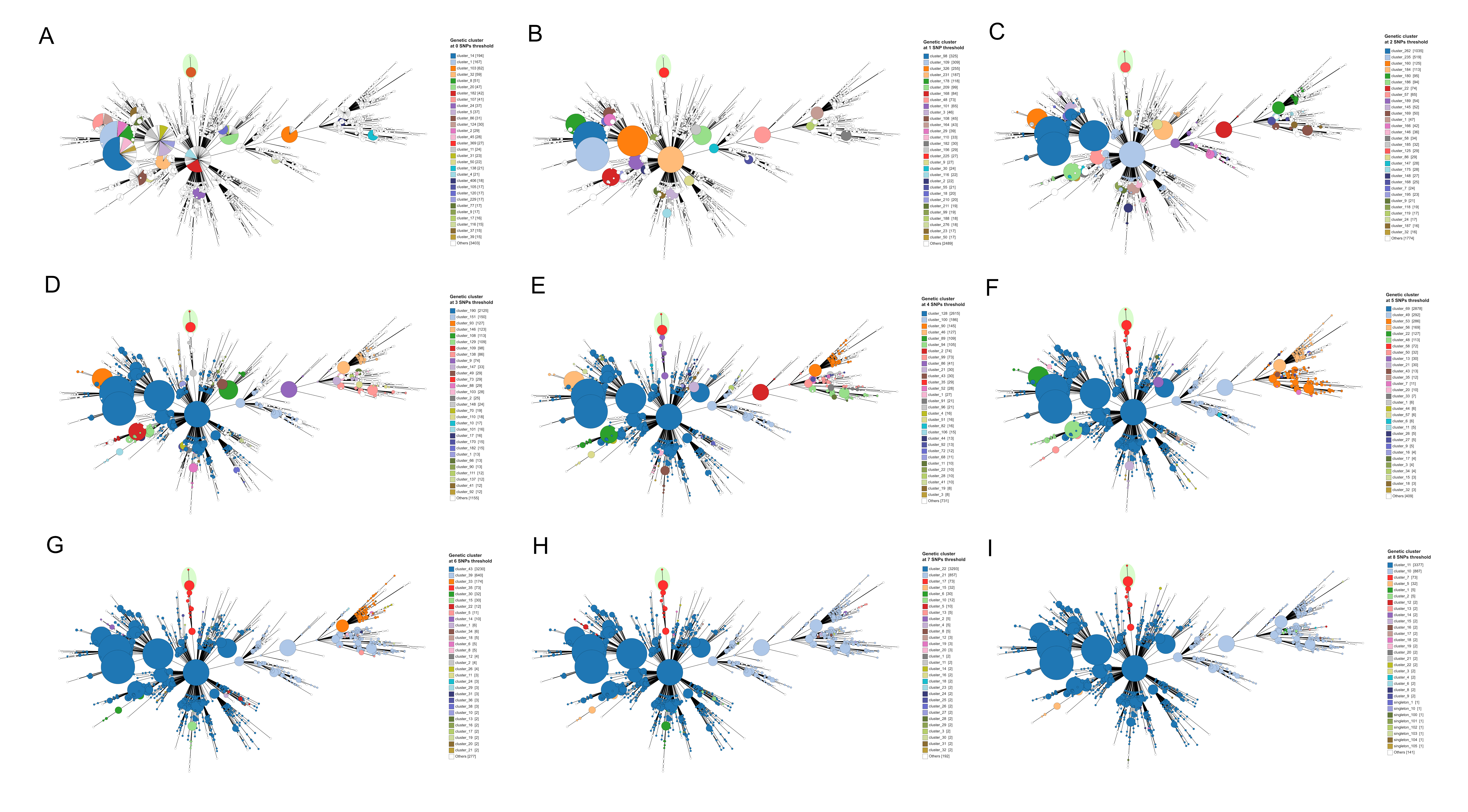

Supplement: Figure_S5.png [file TEMI_A_2640707_SM1123.png]

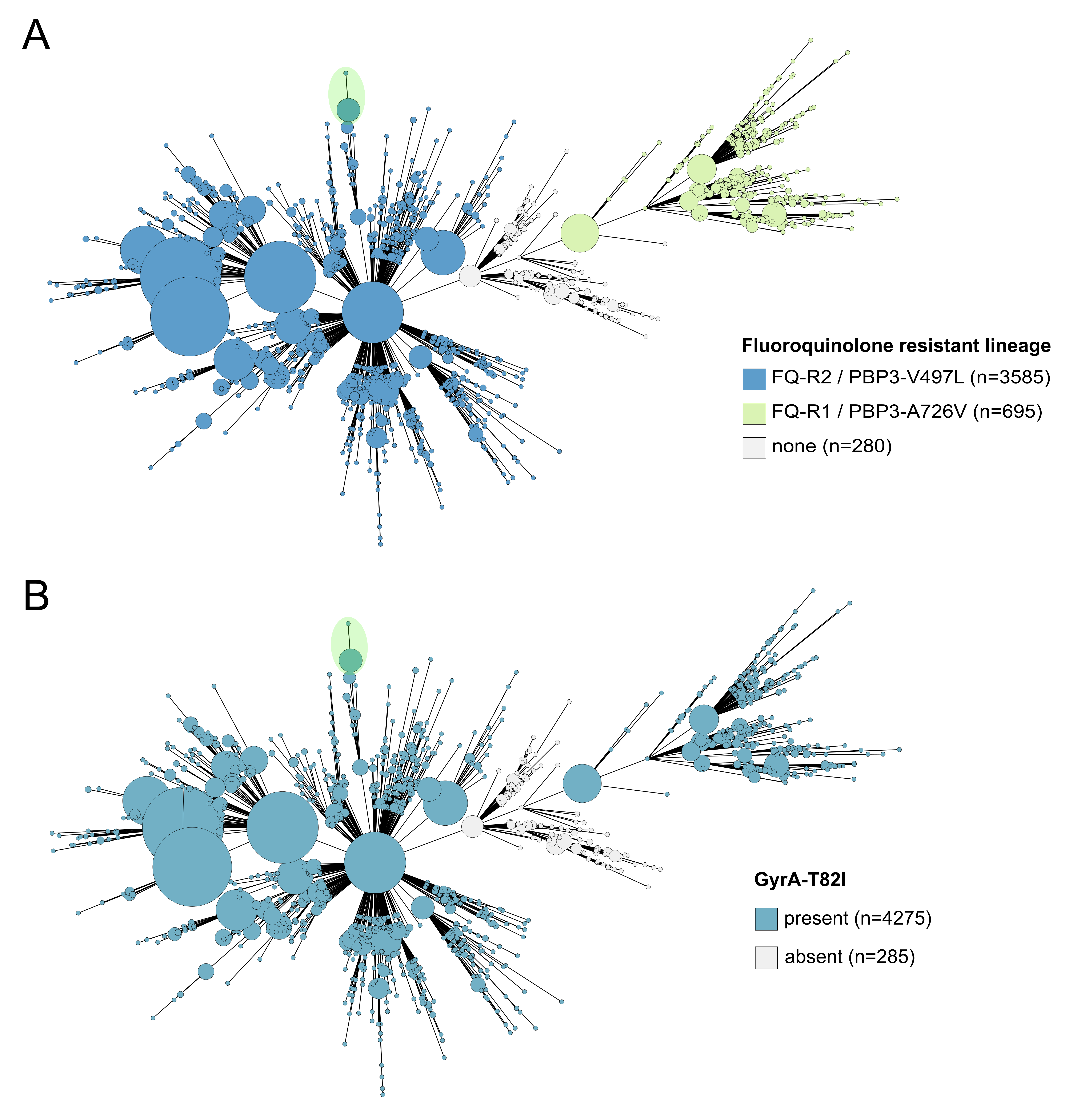

Supplement: Figure_S4_new.png [file TEMI_A_2640707_SM1122.png]

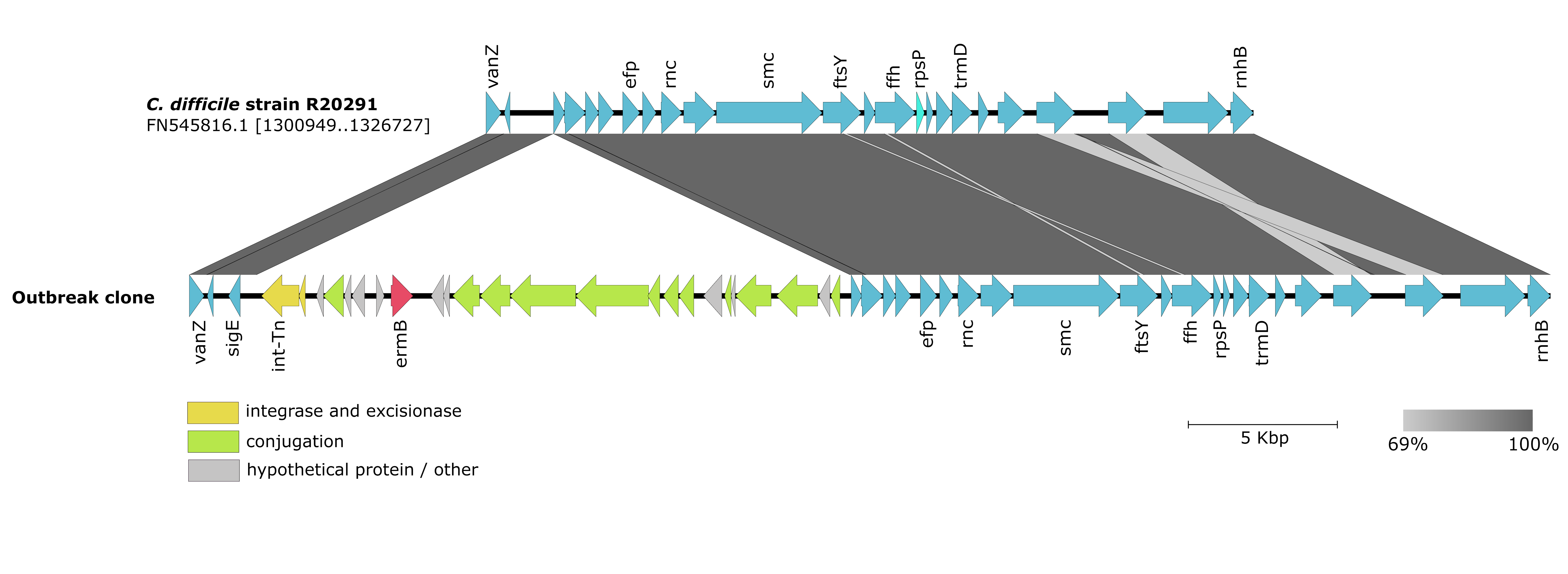

Supplement: Figure S1.png [file TEMI_A_2640707_SM1121.png]
