# Supplementary material for: Genomic evolution and re-emergence of a multidrug-resistant Clostridioides difficile RT027 clone with reduced vancomycin susceptibility driving a prolonged hospital outbreak
Source: Emerg Microbes Infect. 2026 Mar 3;15(1):2640707. doi: 10.1080/22221751.2026.2640707 (PMC12997477; doi:10.1080/22221751.2026.2640707)
Supplement: CDI MDR RT027 Outbreak_PT_Supplementary material_revised.docx [file TEMI_A_2640707_SM1124.docx]

**Supplementary Material**

# Materials and Methods

## Isolation and characterization of Clostridioides difficile

The faecal samples underwent ethanol shock following inoculation onto ChromID® *C. difficile* agar (bioMérieux, Marcy l’Etoile, France) and incubation in anaerobic atmosphere, using Anoxomat, at 37°C for 48 hours. *C. difficile*-suspected colonies were cultured on Brucella blood agar supplemented with hemin and vitamin K1 (BBA) (BD, BBL, Heidelberg, Germany) under anaerobic conditions for 24 h at 37°C, and species was confirmed by MALDI-TOF (VITEK® MS, bioMérieux). Genomic DNA was extracted using the Isolate II Genomic DNA kit (Bioline, London, United Kingdom), according to manufacturer's instructions. Each isolate was characterized by multiplex PCR, targeting *gluD* and the *tcdA, tcdB, cdtA* and *cdtB* toxin genes, according to Persson et al. (2009) [1], and by PCR-ribotyping using Bidet primers [2] followed by capillary gel-based electrophoresis, according to Fawley et al. (2015) [3]. The ribotype profile was identified using the Webribo database (<https://webribo.ages.at/>). Antimicrobial susceptibility testing was performed using Etest^®^ strips (bioMerieux) on BBA. Minimum inhibitory concentrations (MICs) were determined after 24h of incubation under anaerobic conditions. Interpretive categories susceptible (S) or resistant (R) were assigned according to established MIC breakpoints. For vancomycin (>2 mg/L) and metronidazole (>2 mg/L) the European Committee on Antimicrobial Susceptibility Testing (EUCAST) version 15.0 guidelines were applied [4]. Breakpoints for moxifloxacin (≥8 mg/L), clindamycin (≥8 mg/L), rifampicin (≥16 mg/L) and chloramphenicol (≥32 mg/L) were defined according to Freeman et al., (2015) [5]. The Clinical and Laboratory Standards Institute (CLSI) interpretative values for anaerobes were used for imipenem (≥16 mg/L), erythromycin (≥8 mg/L), tetracycline (≥16 mg/L) and linezolid (>4 mg/L). CLSI interpretative values for *Staphylococcus* spp. were used for gentamicin (≥16 mg/L), according to Isidro et al. (2018) [6]. For trimethoprim, interpretive breakpoints for *Staphylococcus* spp. were applied for reference purposes, according to EUCAST (MIC > 4 mg/L).

## Genomic analysis

Genome *de novo* assembly was performed with the INNUca pipeline v4.2.3 (<https://github.com/B-UMMI/INNUca>, which integrates several analysis steps, including i) raw reads quality evaluation (FASTQC v0.11.5; <https://www.bioinformatics.babraham.ac.uk/projects/fastqc/>) and improvement (Trimmomatic v0.39) [7], ii) *de novo* assembly with Spades v3.15.3, iii) post-assembly optimization with Pilon v1.24 [8], iv) species confirmation and detection of contamination with Kraken 2 [9] and v) MLST typing (<https://github.com/tseemann/mlst>).

For in-depth Single nucleotide polymorphism (SNP)-based comparison between outbreak strains and other closely related sequences, variant calling was done with Snippy v4.5.1 (<https://github.com/tseemann/snippy>), with the parameters *--mapqual 30 --mincov 10 --basequal 20*, using the reads after quality control. Core variable positions were extracted using snippy-core and used to generate a maximum likelihood tree with 100 bootstraps with MEGA [10].

For the identification of genetic determinants of antimicrobial resistance and virulence factors, genome assemblies were screened against the databases Resfinder, the Comprehensive Antibiotic Resistance Database (CARD) and the Virulence Factor Database (VFDB) using ABRicate (<https://github.com/tseemann/abricate>). In parallel, to aid the identification of mobile genetic elements, genomes were also analysed using the pipeline Clostyper (<https://gitlab.com/FLI_Bioinfo/clostyper>).

For the integration of the outbreak-associated genomes in the global diversity of *C. difficile*, all the assemblies available in NCBI’s Genbank and RefSeq, as well as through the AllTheBacteria project [11] (<https://allthebacteria.readthedocs.io/en/latest/>) as of August 2024 (latest version) were downloaded and filtered for those with the profile ST1 *cdtA*+/*cdtB*+, as a means to identify those most likely to be RT027. We then applied a set of conservative criteria to filter out genomes with poorer quality, namely we excluded assemblies with i) more than 400 contigs, ii) less than 4 Mb and more than 4.7 Mb in length, iii) N50 below 10 kb, iv) <80% of contigs classified as *Peptostreptococcaceae* at the family level, v) >5% of contigs classified as *Homo sapiens* and vi) multiple copies of the same toxin genes. Also, a single genome was kept for each biosample number, with preference for RefSeq assemblies when available.

A SNP-based approach was then applied for the global dataset analysis. Firstly, variant calling was performed with Snippy v4.5.1 against the reference genome R20291 to obtain a multiple genome alignment. The alignment was then analysed with Reportree [12], with the parameters *--loci-called 0.95 --site-inclusion 0.95 --method MSTreeV2 --analysis grapetree*, that after SNP-matrix extraction, generated a minimum spanning tree (MST) and identified the genetic clusters at all possible SNP thresholds. MST was vizualized with Grapetree [13] and clustering information was used to identify the genomes more closely related to the outbreak clone.

The genomes were screened for mutations in the genes coding for PBP1 and PBP3 that are markers of the RT027 fluoroquinolone resistant lineages FQ-R1 and FQ-R2, as described by Dingle and colleagues [14]

# References

[1] Persson S, Torpdahl M, Olsen KEP. Erratum: New multiplex PCR method for the detection of the *Clostridium difficile* toxin A (tcdA) and toxin B (tcdB) and the binary toxin (cdtA/cdtB) genes applied to a Danish strain collection (Clinical Microbiology and Infection (2008) vol. 14 (1057-1064). Clin Microbiol Infect. 2009;15(3):296.

[2] Bidet P, Barbut F, Lalande V, et al. Development of a new PCR-ribotyping method for *Clostridium difficile* based on ribosomal RNA gene sequencing. FEMS Microbiol Lett. 1999;175(2):261–266.

[3] Fawley WN, Knetsch CW, MacCannell DR, et al. Development and validation of an internationally-standardized, high-resolution capillary gel-based electrophoresis PCR-ribotyping protocol for *Clostridium difficile*. PLoS One. 2015;10(2):1–14.

[4] EUCAST. The European Committee on Antimicrobial Susceptibility Testing. Breakpoint tables for interpretation of MICs and zone diameters. Version 15.0. 2025. Available from: https://www.eucast.org.

[5] Freeman J, Vernon J, Morris K, et al. Pan-European longitudinal surveillance of antibiotic resistance among prevalent *Clostridium difficile* ribotypes. Clin Microbiol Infect. 2015;21(3):248.e9-248.e16.

[6] Isidro J, Santos A, Nunes A, et al. Imipenem resistance in *Clostridium difficile* ribotype 017, Portugal. Emerg Infect Dis. 2018;24(4):741–745.

[7] Bolger AM, Lohse M, Usadel B. Trimmomatic: a flexible trimmer for Illumina sequence data. Bioinformatics. 2014;30(15):2114–2120.

[8] Walker BJ, Abeel T, Shea T, et al. Pilon: an integrated tool for comprehensive microbial variant detection and genome assembly improvement. PLoS One. 2014;9(11):e112963.

[9] Wood DE, Lu J, Langmead B. Improved metagenomic analysis with Kraken 2. Genome Biol. 2019;20(1):257.

[10] Kumar S, Stecher G, Li M, et al. MEGA X: Molecular Evolutionary Genetics Analysis across Computing Platforms. Mol Biol Evol. 2018;35(6):1547–1549.

[11] Hunt M, Lima L, Anderson D, et al. AllTheBacteria – all bacterial genomes assembled, available, and searchable. bioRxiv. 2025;2024.03.08.584059.

[12] Mixão V, Pinto M, Gomes JP, et al. ReporTree: a surveillance-oriented tool to strengthen the linkage between pathogen genetic clusters and epidemiological data. Res Sq. 2022; doi: 10.21203/rs.3.rs-1404655/v1.

[13] Zhou Z, Alikhan N-F, Sergeant MJ, et al. GrapeTree: visualization of core genomic relationships among 100,000 bacterial pathogens. Genome Res. 2018;28(9):1395–1404.

[14] E. DK, Jane F, Xavier D, et al. Penicillin Binding Protein Substitutions Cooccur with Fluoroquinolone Resistance in Epidemic Lineages of Multidrug-Resistant Clostridioides difficile. MBio. 2023;14(2):e00243-23.

Table S1. CDI outbreak summary and cases’ ward distribution.

| **Ward** | **Start of the outbreak** | **End of the outbreak** | **Total CDI cases** | **First CDI episodes** | **Recurrent CDI cases** |
| --- | --- | --- | --- | --- | --- |
| A –Infectious Diseases | 12/10/2023 | 28/12/2023 | 7 | 5 | 2 |
| B – Medicine A | 27/12/2023 | 06/08/2024 | 21 | 16 | 5 |
| C – Medicine B | 08/04/2024 | 21/06/2024 | 8 | 5 | 3 |
| D - Medicine C | 9/04/2024 | 14/06/2024 | 3 | 3 | 0 |
| E - Orthopaedics | 15/05/2024 | 21/07/2024 | 3 | 3 | 0 |
| F – Medicine D | 16/07/2024 | 17/11/2024 | 7 | 6 | 1 |
| G – Medicine E | 2/10/2024 | 31/01/2025 | 6 | 5 | 1 |
| Other | - | - | 11 | 9 | 2 |
| Total | - | - | 66 | 52 | 14 |

Notes: Medicine refers to General internal medicine wards;

CDI cases that were not attributable to any of the main outbreak wards were classified as “Other” and grouped together, comprising Nephrology (2), Orthopaedics - another ward (2), General internal medicine - another wards (5).

Table S2 - First-episode CDI antimicrobial therapy regimens

| **Antimicrobial regimen** | **Number of patients**  **(n; %)** | **Duration**  **(range in number of days; ATB _1_ / ATB _2_ / ATB _n_)** | **Recurrence**  **(n; %)** |
| --- | --- | --- | --- |
| Vancomycin | 34; 65.4% | 1 – 45 | 12*; 35.3% |
| Vancomycin followed by fidaxomicin | 10; 19.2%; | (2 – 13) / (9 – 20) | 1; 10% |
| Fidaxomicin | 2; 3.9% | 10 | 0 |
| Vancomycin followed by vancomycin with metronidazole | 2; 3.9% | (2 – 10) / (3 – 10) | 0 |
| Vancomycin with metronidazole | 1; 1.9% | 10 | 0 |
| Fidaxomicin followed by vancomycin | 1; 1.9% | 18 / 10 | 0 |
| Fidaxomicin, followed by vancomycin, metronidazole, and fidaxomicin. | 1; 1.9% | 2 / 7 / 3 / 2 | 0 |
| Vancomycin, followed by fidaxomicin with tigecycline and extended duration vancomycin | 1; 1.9% | 10 / 19 + 4 / 43 | 0 |

* Eleven first recurrences and one second recurrence

# Figures

# Figure captions

Figure S1. Structure and genomic context of the Tn6189-like transposon containing ermB. Genetic content of the 20 kb Tn6189-like mobile genetic element identified in the outbreak clone that contains the MLSb resistance gene ermB. The genomic location of the transposon is shown by comparison with the reference genome of C. difficile strain R20291 (FN545816.1). The figure was generated with Easyfig v2.2.5 and finalised with Inkscape v1.4.2.

Figure S2. Structure and genomic context of the putative element containing dfrF. Characterization of the 6.5 kb region containing the dfrF gene. The genomic location of the putative MGE is shown by comparison with the reference genome of C. difficile strain R20291 (FN545816.1). The figure was generated with Easyfig v2.2.5 and finalised with Inkscape v1.4.2.

Figure S3. Genetic content of the mobile element harbouring the *cfrB* gene. Characterization of the 10.5 kb *cfrB*-containing mobile genetic element (MGE) that was found in the outbreak clone. As this MGE is also found in other publicly available (and annotated) genomes closely related to the outbreak clone, the MGE sequence in strain LC3P-124-8 (assembly GCF_007002145.1, see Figure X) is shown here. A highly similar MGE was found in the genome of *Enterococcus faecium* strain DB-1; this MGE has high homology to the whole sequence with the exception of the *cfrB* gene region that is absent in *E. faecium* and that seems to have been inserted interrupting a permease coding gene. The figure was generated with Easyfig v2.2.5 and finalised with Inkscape v1.4.2.

Figure S4. Minimum spanning tree (MST) of a global dataset of RT027 genomes coloured by fluoroquinolone resistant lineage and GyrA T82I mutation. The MST was generated with Reportree based on 8065 core variant positions extracted from a multiple genome alignment of 4560 sequences with 4191339 bp generated with Snippy-core after mapping against the *C. difficile* strain R20291 reference genome (see methods for details). The MST was visualised in Grapetree with branches collapsed at 1 SNP threshold. MST nodes are coloured according to A) the fluorquinolone resistant (FQ) lineage, identified by the respective marker mutations in PBP3 (as described by XXX) and B) the presence of the substitution T82I in GyrA, associated with fluoquinolones resistance. The outbreak-associated sequences are highlighted by a green ellipse in each panel. Note that due to the filtering criteria applied when running Reportree, some genomes are excluded from the MST.

Figure S5. Minimum spanning tree (MST) of a global dataset of RT027 genomes coloured by genetic cluster at different SNP thresholds. The MST was generated with Reportree based on 8065 core variant positions extracted from a multiple genome alignment of 4560 sequences with 4191339 bp generated with Snippy-core after mapping against the *C. difficile* strain R20291 reference genome (see methods for details). The MST was visualised in Grapetree with branches collapsed at 1 SNP threshold. MST nodes are coloured according to the genetic clusters identified by Reportree at increasing SNP thresholds, specifically from 0 to 8 SNPs, respectively corresponding to panels A) to I), showing the Portuguese outbreak-associated sequences highlighted by a green ellipse in each panel. Note that due to the filtering criteria applied when running Reportree, some genomes are excluded from the MST.
